# Severe Skew Foot Deformity in a Patient With Freeman-Sheldon Syndrome

**DOI:** 10.4021/jocmr653e

**Published:** 2011-09-26

**Authors:** Ali Al Kaissi, Klaus Klaushofer, Franz Grill

**Affiliations:** a Ludwig Boltzmann Institute of Osteology, Hanusch Hospital of WGKK and AUVA Trauma Centre Meidling, First Medical Department, Hanusch Hospital, Vienna, Austria; b Orthopaedic Hospital of Speising, Paediatric Department, Vienna, Austria

## Abstract

We report on a 3-year-old boy with the full phenotypic features of Freeman Sheldon syndrome (FSS). Severe skew foot deformity has been recognized as additional skeletal abnormality. Parents were first degree cousins, raising the possibility of autosomal recessive pattern of inheritance. To the best of our knowledge this is the first report of severe skew foot deformity in a patient with (FSS).

**Keywords:**

Freeman-Sheldon syndrome; Skew foot deformity; Metatarsus adductus

## Introduction

Craniocarpotarsal syndrome was first described in 1938 by Freeman, an orthopaedic surgeon, and Sheldon, a paediatrician. Inheritance of FSS is usually sporadic, but both autosomal dominant and autosomal recessive transmission have been reported [1-3].

FSS is characterized by a typical ”whistling“ facies consisting of a small, pursed mouth; long philtrum, small nose, deeply sunken eyes and scar like contracture that extends from the middle of the lower lip to the chin. In addition patients exhibit ulnar deviation and thumb-in-palm deformity. Other associated anomalies are scoliosis and kyphoscoliosis, developmental dislocation of the hip, occasional spina bifida occulta, asymmetric pinnae, mild petrygium coli, and pectus excavatum [4]. The clinical presentation may mimic that of distal arthrogryposis [5]. Rigid talipes equinovarus and vertical talus have been considered as the most frequent foot deformities in connection with (FSS). Skew foot is a condition that resembles metatarsus adductus but the elements of adductus of the forefoot and valgus of the hindfoot are more severe and rigid [6].

## Case Report

A 3-year-old boy was referred to our department for clinical assessment. Growth has been retarded below the third percentile, the facial skeleton is small; facial characteristics include deep-sunken eyes with hypertelorism, increased philtrum length, small nose and nostrils, and a small mouth which is difficult to open. The lips are in the ”whistling position“. Highly arched palate, small tongue and a skin dimple on the chin in the shape of an inverted V. Apparently, there is a major orofacial malformation which warrants specialised orthodontic intervention. The limbs are thin with poor musculature and mobility (especially the scapulohumeral joint and pronation-supination of the forearm) were present. Flexed fingers (mostly over the metacarpophalangeal joints) associated with ulnar deviation (wind swept deformity) were notable. Examination of the foot revealed severe forefoot adduction, lateral subluxation (abduction) at the talonavicular joint and valgus of the heel. The deformity was extremely rigid. His vision, hearing and neurological examination were normal though his language skills were limited associated with defective social behaviour. The child had normal genitalia. All other investigations including an abdominal ultrasound, karyotyping, and metabolic tests, which aimed to test calcium, phosphorus, and vitamin D metabolism, were normal. Family history showed first degree cousins healthy parents. The mother had a history of multiple spontaneous abortions and the reason behind was unknown.

**Figure 1:**
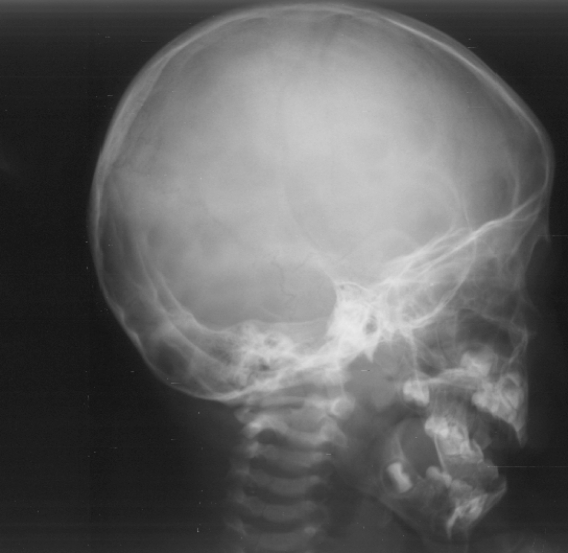
Lateral skull radiograph showed significant hyperostosis of the skull base associated with total sclerosis of the lambdoid sutures and partial sclerosis of the coronal sutures respectively. The anteroposterior length of both base and cranium are relatively shorter than that of the facial height.

**Figure 2:**
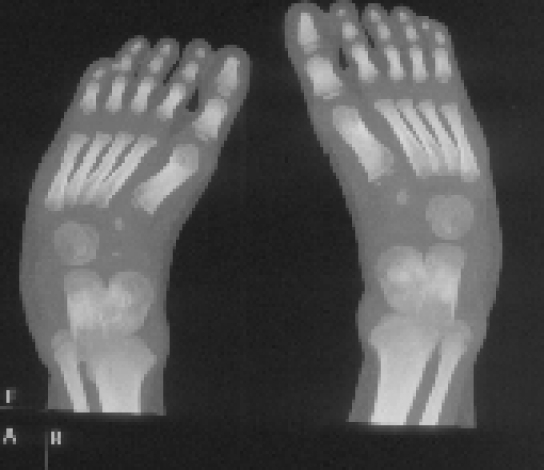
Coronal reformatted CT scan showed a line drawn to the head of the talus and through the body of the talus, makes a Z shape.

**Figure 3:**
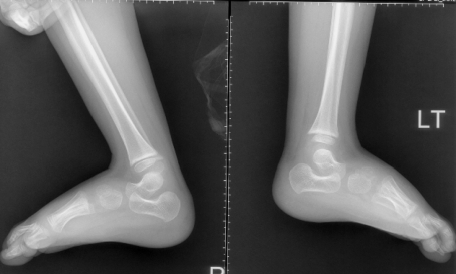
Lateral view showed an increased talocalcaneal angle indicative of valgus of the hindfoot ( there is dorsiflexion of the talonavicular joint probably secondary to increased talar plantar flexion) and plantar flexion of the tarsometatarsal joints, another Z deformity.

Radiographic findings, lateral skull radiograph showed significant hyperostosis of the skull base associated with total sclerosis of the lambdoid sutures and partial sclerosis of the coronal sutures respectively. The anteroposterior length of both base and cranium are relatively shorter than that of the facial height (Fig. 1). Lateral view showed an increased talocalcaneal angle indicative of valgus of the hindfoot (there is dorsiflexion of the talonavicular joint (probably secondary to increased talar plantar flexion) and plantar flexion of the tarsometetarsal joints, another Z deformity (Fig. 2). Coronal reformatted CT scan showed a line drawn to the head of the talus and through the body of the talus, makes a Z shape (Fig. 3).

## Treatment

Because of lack of criteria for definition of the deformity and its relative severity, early treatment of skew-foot is often based on a perception of need to treat the metatarsus adductus. Thus, a more severe adductus that is not passively correctable and is rigid as in our patient, this can be treated by stretching and serial casting. The cast technique must carefully mold the hindfoot into varus to avoid exacerbating the existing valgus while correcting the forefoot. Therefore, we used redression casts in order to get the hindfoot into varus, and to avoid the exacerbating existing valgus deformity. Furthermore we aimed to give a counter pressure on the lateral side of the foot (cuboid bone) to avoid the lateral subluxation of the chopard joint.

## Discussion

In 1938, Freeman and Sheldon [1] described a syndrome characterized by microstomia, flat mid-face, talipes equinovarus, and ulnar deviation of the fingers. Burian [4] described the same complex, utilizing the term ”Whistling face syndrome“. Although most cases have been sporadic, there are several examples of the disorder in two or more generations. The condition has also been described in sibs with normal, in some cases, consanguineous parents.

Hall et al. [7] found approximately 15% of patients with FSS had limited movement of the hips and 5% limitation of movement at the elbow (dislocation of the radial heads has been described). Vanek et al. [8] presented evidence that FSS should be considered a form of myopathic arthrogryposis. Krakowiak et al. [9] reported a large family with distal arthrogryposis, but with a distinctive facial appearance that they felt resembled Freeman-Sheldon syndrome. This was characterised by a triangular face, prominent nasolabial folds, downslanting palpebral fissures, a small mouth, and a prominent chin. Skew foot deformity has not been described in any of the above mentioned reports.

Ng et al. [10] showed that candidate genes for Mendelian disorders can be identified by exome sequencing of a small number of unrelated, affected individuals. Based on the 4 patients, they were able to identify the MYH3 gene as the only candidate gene. With 3 affected individuals, they narrowed the candidates to 2 possible genes. These results suggested that targeted capture and massively parallel sequencing of exomes will be a powerful tool to identify the genetic cause of rare Mendelian disorders.

Four clinical types of skewfoot have been considered; congenital, neurogenic, iatrogenic and idiopathic. Congenital skewfoot associated with syndromic entities such as Larsen syndrome, diastrophic dysplasia, Proteus syndrome and osteogenesis imperfecta are characterized by being very rigid and not corrected by nonoperative measures [11].

## Conclusions

Skew foot is a rare condition that is often missed early in a child's development. Mild and flexible forms can be successfully treated with cast immobilization and shoe therapy. In more severe forms, surgical intervention is indicated if there are underlying neuromuscular conditions or the individual is affected on a daily basis because of the deformity. Careful evaluation and proper surgical procedures selection can realign the foot, resulting in favourable long-term outcomes. Finally we wish to stress that, skew foot is a condition that resembles metatarsus adductus but the elements of adductus of the forefoot and valgus of the hindfoot are more severe and rigid. In addition, there is lateral subluxation of the navicular on the talus. Skew foot is also called the S-shaped foot, the serpentine foot, or the Z-foot. A similar deformity is sometimes seen in a clubfoot that has undergone inadequate midfoot release and excessive hindfoot release.

## References

[1] Freeman EA, Sheldon JH (1938). Cranio-carpo-tarsal dystrophy. Arch Dis Child.

[2] Carakushansky G, Paiva IS, Kahn E, Ribeiro MG (2001). [Recessive type of Freeman-Sheldon syndrome - report of two affected siblings]. J Pediatr.

[3] Fitzsimmons JS, Zaldua V, Chrispin AR (1984). Genetic heterogeneity in the Freeman-Sheldon syndrome: two adults with probable autosomal recessive inheritance. J Med Genet.

[4] Burian F (1963). The "whistling face" characteristic in a compound cranio-facio-corporal syndrome. Br J Plast Surg.

[5] Schrander-Stumpel C, Fryns JP, Beemer FA, Rive FA (1991). Association of distal arthrogryposis, mental retardation, whistling face, and Pierre Robin sequence: evidence for nosologic heterogeneity. Am J Med Genet.

[6] Kite JH (1967). Congenital metatarsus varus. J Bone Joint Surg Am.

[7] Hall JG, Reed SD, Greene G (1982). The distal arthrogryposes: delineation of new entities--review and nosologic discussion. Am J Med Genet.

[8] Vanek J, Janda J, Amblerova V, Losan F (1986). Freeman-Sheldon syndrome: a disorder of congenital myopathic origin. J Med Genet.

[9] Krakowiak PA, Bohnsack JF, Carey JC, Bamshad M (1998). Clinical analysis of a variant of Freeman-Sheldon syndrome (DA2B). Am J Med Genet.

[10] Ng SB, Turner EH, Robertson PD, Flygare SD, Bigham AW, Lee C, Shaffer T (2009). Targeted capture and massively parallel sequencing of 12 human exomes. Nature.

[11] Napiontek M (2002). Skewfoot. J Pediatr Orthop.

